# Dimethyl 7a-carbonyl-2-meth­oxy-7a,7a-bis­(triphenyl­phosphino)-7a-ruthena-1-benzofuran-4,7-dicarboxyl­ate

**DOI:** 10.1107/S1600536808040944

**Published:** 2008-12-13

**Authors:** George R. Clark, Warren R. Roper, Deborah M. Tonei, L. James Wright

**Affiliations:** a Department of Chemistry, The University of Auckland, Private Bag 92019, Auckland, New Zealand

## Abstract

The crystal structure of the title compound, [Ru(C_12_H_12_O_6_)(C_18_H_15_P)_2_(CO)], confirms its formulation as a ruthenabenzofuran, with a slightly distorted octa­hedral coordination environment at the Ru^II^ ion, and mutually *trans* triphenyl­phosphine ligands. The metallabicyclic ring system is essentially planar (maximum deviation 0.059 Å).

## Related literature

For the synthesis and properties of metallabenzenes, see: Bleeke (2001 ▶); Landorf & Haley (2006 ▶); Wright (2006 ▶). For the synthesis and properties of metallabenzeno­ids, see: Paneque *et al.* (2003 ▶); Clark *et al.* (2006 ▶); Yamazaki & Aoki (1976 ▶); Bruce *et al.* (2000 ▶); Clark *et al.* (2008 ▶).
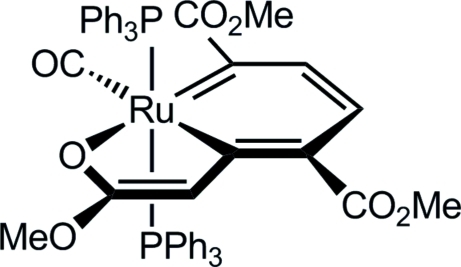


## Experimental

### 

#### Crystal data

[Ru(C_12_H_12_O_6_)(C_18_H_15_P)_2_(CO)]
*M* *_r_* = 905.89
Triclinic, 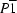

*a* = 12.1102 (5) Å
*b* = 13.2229 (5) Å
*c* = 13.4273 (5) Å
α = 97.746 (1)°
β = 102.616 (1)°
γ = 93.333 (1)°
*V* = 2070.54 (14) Å^3^
*Z* = 2
Mo *K*α radiation
μ = 0.51 mm^−1^
*T* = 85 (2) K
0.28 × 0.22 × 0.20 mm

#### Data collection

Siemens SMART CCD diffractometer
Absorption correction: multi-scan (*SADABS*; Sheldrick, 1996 ▶) *T*_min_ = 0.806, *T*_max_ = 0.921
20010 measured reflections
8434 independent reflections
6871 reflections with *I* > 2σ(*I*)
*R*_int_ = 0.028

#### Refinement

*R*[*F* ^2^ > 2σ(*F* ^2^)] = 0.032
*wR*(*F* ^2^) = 0.071
*S* = 1.04
8434 reflections
532 parameters
H-atom parameters constrained
Δρ_max_ = 0.44 e Å^−3^
Δρ_min_ = −0.51 e Å^−3^

### 

Data collection: *SMART* (Siemens, 1995 ▶); cell refinement: *SAINT* (Siemens, 1995 ▶); data reduction: *SAINT*; program(s) used to solve structure: *SHELXS97* (Sheldrick, 2008 ▶); program(s) used to refine structure: *SHELXL97* (Sheldrick, 2008 ▶); molecular graphics: *ORTEPIII* (Burnett & Johnson, 1996 ▶); software used to prepare material for publication: *SHELXTL* (Sheldrick, 2008 ▶).

## Supplementary Material

Crystal structure: contains datablocks global, I. DOI: 10.1107/S1600536808040944/lh2738sup1.cif

Structure factors: contains datablocks I. DOI: 10.1107/S1600536808040944/lh2738Isup2.hkl

Additional supplementary materials:  crystallographic information; 3D view; checkCIF report

## Figures and Tables

**Table 1:** Selected bond lengths (Å)

Ru—C13	1.907 (2)
Ru—C1	2.038 (2)
Ru—C5	2.110 (2)
Ru—O6	2.2164 (16)
Ru—P1	2.3796 (6)
Ru—P2	2.3919 (6)

**Fig. 1.**
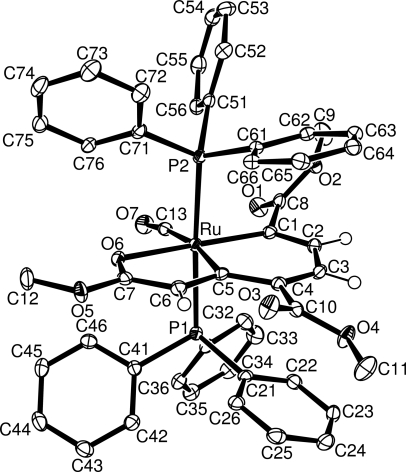
: The molecular structure showing 50% probability displacement ellipsoids for non-hydrogen atoms and selected hydrogen atoms as arbitary spheres (Burnett & Johnson, 1996).

**Fig. 2.**
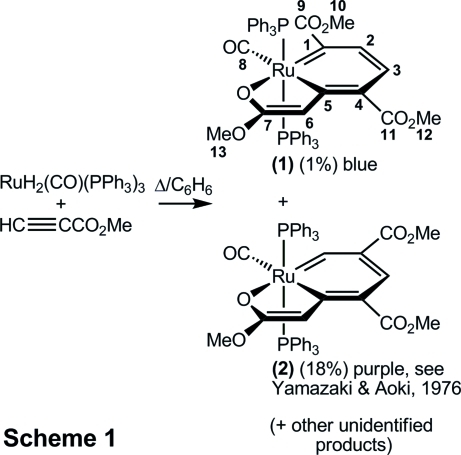
: The reaction scheme.

## supplementary crystallographic information

### Comment

Metallabenzenes are now a well established class of organometallic compounds and a considerable number of studies involving the syntheses, reactivity, aromatic character and decomposition pathways of these materials have been made (Bleeke, 2001, Landorf & Haley, 2006, Wright, 2006). In contrast, studies of fused ring metallabenzenoids such as metallanaphthalenes (Paneque *et al.*, 2003) and metallabenzofurans (Clark *et al.*, 2006) are much more rare. We recently reported that protonation of a carbon atom in the five-membered ruthenafuran ring of the known ruthenabenzofuran, Ru[C_5_H_2_(CO_2_Me-2)(CO_2_Me-4)(CHCO_2_Me-5)](CO)(PPh_3_)_2_(2) (see Fig. 2) (Yamazaki & Aoki, 1976, Bruce *et al.*, 2000) provides a new route to ruthenabenzenes (Clark *et al.*, 2009). While following the literature synthesis of this ruthenabenzofuran, (Yamazaki & Aoki, 1976), we were able to isolate through chromatography a small amount of a previously unreported, isomeric ruthenabenzofuran, Ru[C_5_H_2_(CO_2_Me-1)(CO_2_Me-4)(CHCO_2_Me-5)](CO)(PPh_3_)_2_ (1) (see Fig. 2). We now report details of the structure of (1) (Fig. 1) which confirms its formulation as a ruthenabenzofuran, with essentially octahedral coordination at Ru, and mutually *trans* triphenylphosphine ligands. The metallabicyclic ring system is essentially planar. The C—C bond lengths within the six-membered ring show a small but significant alternation, similar to that reported in the isomeric ruthenabenzofuran (Bruce *et al.*, 2000) in which one of the methyl ester substituents resides on C2 rather than C1. This change has no important impact on the structural parameters of the metallabicyclic ring system. The bond length alternations in these two ruthenabenzofuran isomers are more pronounced than in the tethered ruthenabenzene derived from the isomer reported by Bruce (Bruce *et al.*, 2000) by protonation at C6 (Clark *et al.*, 2009).

### Experimental

RuH_2_(CO)(PPh_3_)_3_ (1.00 g, 1.09 mmol) and methyl propiolate (0.55 g, 0.58 ml, 6.54 mmol) were heated under reflux in benzene (50 ml) for one hour. The initially pale yellow-green solution changed to red-brown soon after the solution reached boiling point. The solvent was removed under vacuum and the residue purified by chromatography on silica gel using dichloromethane/ethanol (98:2) as eluent. Two coloured bands were eluted from the column. The first band, which was coloured blue, was collected and on evaporation of the solvent dark blue crystals of the title compound were obtained (0.0098 g, 1%). The second, much larger red-purple band contained the related ruthenabenzofuran, Ru[C_5_H_2_(CO_2_Me-2)(CO_2_Me-4)(CHCO_2_Me-5)](CO)(PPh_3_)_2_ (2), previously reported in the literature (Yamazaki & Aoki, 1976). The crystal of Ru[C_5_H_2_(CO_2_Me-1)(CO_2_Me-4)(CHCO_2_Me-5)](CO) (PPh_3_)_2_ (1) that was used for the single-crystal X-ray diffraction study was grown from dichloromethane/ethanol solution. The atom numbering used for NMR assignments is given in Fig. 2. ^1^H NMR (CDCl_3_, δ p.p.m., TMS = 0.00), 7.10 - 7.75 (*m*, 30H, PPh_3_), 6.58 (*dt*, 1H, ^4^*J*_HH_ = 8.1 Hz, ^4^*J*_HP_ = 1.9 Hz, H2), 6.53 (*d*, 1H, ^4^*J*_HH_ = 8.1 Hz, H3), 6.02 (*t*, 1H, ^4^*J*_HP_ = 3.5 Hz, H6), 3.59 (*s*, 3H, CO_2_C*H*_3_, H10), 3.33 (*s*, 3H, CO_2_C*H*_3_, H12), 3.18 (*s*, 3H, CO_2_C*H*_3_, H13). ^13^C{^1^H} NMR (CDCl_3_, δ p.p.m., TMS = 0.00), 225.8 (^2^*J*_CP_ = 13.6 Hz, C5), 205.3 (^2^*J*_CP_ = 13.1 Hz, *C*O, C8), 194.9 (*t*, ^2^*J*_CP_ = 13.1, C1), 179.4 (*s*, *C*O_2_Me, C7), 175.8 (*s*, *C*O_2_Me, C11), 168.0 (*s*, *C*O_2_Me, C9), 147.5 (*s*, *C*H, C3), 126–135 (*m*, PPh_3_), 129.6 (*s*, *C*H, C2), 124.4 (*s*, C4), 121.0 (*t*, *C*H, ^3^*J*_CP_ = 4.5, C6), 51.9 (*s*, CO_2_*C*H_3_, C13), 51.2 (*s*, CO_2_*C*H_3_, C12), 50.5 (*s*, CO_2_*C*H_3_, C10). ^31^P{^1^H} NMR (CDCl_3_, δ p.p.m., 85% orthophosphoric acid external std. = 0.00), 39.27 (*s*).

### Refinement

Hydrogen atoms were placed in calculated positions and refined using the riding model [C—H 0.93–0.97 Å), with *U*_iso_(H) = 1.2 or 1.5 times *U*_eq_(C).

### Crystal data

**Table:**

[Ru(C_12_H_12_O_6_)(C_18_H_15_P)_2_(CO)]	*Z* = 2
*M**_r_* = 905.89	*F*(000) = 932
Triclinic, *P*1	*D*_x_ = 1.453 Mg m^−^^3^
Hall symbol: -p 1	Mo *K*α radiation, λ = 0.71073 Å
*a* = 12.1102 (5) Å	Cell parameters from 5960 reflections
*b* = 13.2229 (5) Å	θ = 1.6–26.4°
*c* = 13.4273 (5) Å	µ = 0.51 mm^−^^1^
α = 97.746 (1)°	*T* = 85 K
β = 102.616 (1)°	Block, purple
γ = 93.333 (1)°	0.28 × 0.22 × 0.20 mm
*V* = 2070.54 (14) Å^3^	

### Data collection

**Table:**

Siemens SMART CCD diffractometer	8434 independent reflections
Radiation source: fine-focus sealed tube	6871 reflections with *I* > 2σ(*I*)
graphite	*R*_int_ = 0.028
Area detector ω scans	θ_max_ = 26.4°, θ_min_ = 1.6°
Absorption correction: multi-scan (*SADABS*; Sheldrick, 1996)	*h* = −15→14
*T*_min_ = 0.807, *T*_max_ = 0.921	*k* = −16→16
20010 measured reflections	*l* = 0→16

### Refinement

**Table:**

Refinement on *F*^2^	Primary atom site location: structure-invariant direct methods
Least-squares matrix: full	Secondary atom site location: difference Fourier map
*R*[*F*^2^ > 2σ(*F*^2^)] = 0.032	Hydrogen site location: inferred from neighbouring sites
*wR*(*F*^2^) = 0.071	H-atom parameters constrained
*S* = 1.04	*w* = 1/[σ^2^(*F*_o_^2^) + (0.022*P*)^2^ + 1.4641*P*] where *P* = (*F*_o_^2^ + 2*F*_c_^2^)/3
8434 reflections	(Δ/σ)_max_ < 0.001
532 parameters	Δρ_max_ = 0.44 e Å^−^^3^
0 restraints	Δρ_min_ = −0.51 e Å^−^^3^

### Special details

**Table:**

Geometry. All e.s.d.'s (except the e.s.d. in the dihedral angle between two l.s. planes) are estimated using the full covariance matrix. The cell e.s.d.'s are taken into account individually in the estimation of e.s.d.'s in distances, angles and torsion angles; correlations between e.s.d.'s in cell parameters are only used when they are defined by crystal symmetry. An approximate (isotropic) treatment of cell e.s.d.'s is used for estimating e.s.d.'s involving l.s. planes.
Refinement. Refinement of *F*^2^ against ALL reflections. The weighted *R*-factor *wR* and goodness of fit *S* are based on *F*^2^, conventional *R*-factors *R* are based on *F*, with *F* set to zero for negative *F*^2^. The threshold expression of *F*^2^ > σ(*F*^2^) is used only for calculating *R*-factors(gt) *etc*. and is not relevant to the choice of reflections for refinement. *R*-factors based on *F*^2^ are statistically about twice as large as those based on *F*, and *R*- factors based on ALL data will be even larger.

### Fractional atomic coordinates and isotropic or equivalent isotropic displacement parameters (Å^2^)

**Table:**

	*x*	*y*	*z*	*U*_iso_*/*U*_eq_	
Ru	0.216450 (16)	0.201239 (14)	0.215449 (14)	0.01165 (6)	
P1	0.15355 (5)	0.11361 (4)	0.33937 (4)	0.01214 (13)	
P2	0.27620 (5)	0.30884 (4)	0.10386 (4)	0.01183 (13)	
O1	0.46834 (15)	0.07527 (12)	0.23988 (13)	0.0231 (4)	
O2	0.56152 (14)	0.23144 (12)	0.26121 (13)	0.0197 (4)	
O3	0.12784 (15)	0.53793 (13)	0.40538 (13)	0.0232 (4)	
O4	0.26131 (15)	0.51234 (13)	0.54176 (12)	0.0210 (4)	
O5	−0.11237 (14)	0.29881 (13)	0.14179 (13)	0.0194 (4)	
O6	0.03690 (13)	0.20657 (12)	0.13497 (11)	0.0143 (3)	
O7	0.23080 (15)	−0.01145 (13)	0.10036 (13)	0.0244 (4)	
C1	0.3802 (2)	0.22584 (17)	0.29898 (17)	0.0136 (5)	
C2	0.4191 (2)	0.30178 (17)	0.38134 (17)	0.0148 (5)	
H2	0.4956	0.3043	0.4136	0.018*	
C3	0.3557 (2)	0.37805 (17)	0.42395 (17)	0.0151 (5)	
H3	0.3942	0.4191	0.4849	0.018*	
C4	0.2460 (2)	0.39826 (17)	0.38648 (17)	0.0133 (5)	
C5	0.1714 (2)	0.33467 (17)	0.29668 (17)	0.0133 (5)	
C6	0.0607 (2)	0.35233 (17)	0.26249 (17)	0.0150 (5)	
H6	0.0290	0.4073	0.2931	0.018*	
C7	−0.0038 (2)	0.28123 (18)	0.17665 (17)	0.0153 (5)	
C8	0.4716 (2)	0.16743 (18)	0.26509 (17)	0.0164 (5)	
C9	0.6523 (2)	0.1826 (2)	0.2262 (2)	0.0304 (7)	
H9A	0.7119	0.2337	0.2260	0.046*	
H9B	0.6241	0.1462	0.1576	0.046*	
H9C	0.6815	0.1355	0.2718	0.046*	
C10	0.2040 (2)	0.48872 (17)	0.44205 (18)	0.0151 (5)	
C11	0.2193 (2)	0.5936 (2)	0.60212 (19)	0.0273 (6)	
H11A	0.2649	0.6051	0.6714	0.041*	
H11B	0.1419	0.5746	0.6031	0.041*	
H11C	0.2232	0.6552	0.5720	0.041*	
C12	−0.1756 (2)	0.2300 (2)	0.05143 (19)	0.0243 (6)	
H12A	−0.2517	0.2492	0.0329	0.036*	
H12B	−0.1775	0.1610	0.0665	0.036*	
H12C	−0.1393	0.2341	−0.0049	0.036*	
C13	0.2325 (2)	0.07083 (18)	0.14256 (18)	0.0152 (5)	
C21	0.1726 (2)	0.18875 (17)	0.46847 (17)	0.0139 (5)	
C22	0.2708 (2)	0.18608 (18)	0.54457 (18)	0.0170 (5)	
H22	0.3264	0.1442	0.5307	0.020*	
C23	0.2864 (2)	0.24553 (18)	0.64120 (18)	0.0203 (5)	
H23	0.3522	0.2428	0.6913	0.024*	
C24	0.2055 (2)	0.30838 (19)	0.66338 (19)	0.0233 (6)	
H24	0.2157	0.3467	0.7286	0.028*	
C25	0.1084 (2)	0.31427 (18)	0.58767 (19)	0.0203 (5)	
H25	0.0543	0.3577	0.6018	0.024*	
C26	0.0922 (2)	0.25548 (17)	0.49108 (18)	0.0160 (5)	
H26	0.0273	0.2603	0.4407	0.019*	
C31	0.2151 (2)	−0.00574 (17)	0.37078 (17)	0.0146 (5)	
C32	0.3329 (2)	−0.00963 (19)	0.38983 (19)	0.0204 (5)	
H32	0.3787	0.0449	0.3790	0.024*	
C33	0.3820 (2)	−0.09495 (19)	0.4250 (2)	0.0242 (6)	
H33	0.4605	−0.0964	0.4387	0.029*	
C34	0.3147 (2)	−0.17755 (18)	0.43951 (19)	0.0217 (6)	
H34	0.3480	−0.2334	0.4646	0.026*	
C35	0.1978 (2)	−0.17655 (18)	0.41653 (18)	0.0203 (5)	
H35	0.1523	−0.2329	0.4239	0.024*	
C36	0.1480 (2)	−0.09131 (18)	0.38229 (17)	0.0180 (5)	
H36	0.0694	−0.0913	0.3669	0.022*	
C41	0.0027 (2)	0.06660 (17)	0.30360 (17)	0.0141 (5)	
C42	−0.0622 (2)	0.05808 (18)	0.37681 (18)	0.0168 (5)	
H42	−0.0323	0.0867	0.4453	0.020*	
C43	−0.1705 (2)	0.00757 (18)	0.34865 (19)	0.0212 (5)	
H43	−0.2127	0.0024	0.3982	0.025*	
C44	−0.2159 (2)	−0.03522 (19)	0.24688 (19)	0.0220 (6)	
H44	−0.2884	−0.0693	0.2281	0.026*	
C45	−0.1530 (2)	−0.02715 (18)	0.17315 (19)	0.0196 (5)	
H45	−0.1838	−0.0555	0.1047	0.023*	
C46	−0.0442 (2)	0.02310 (17)	0.20093 (18)	0.0165 (5)	
H46	−0.0024	0.0278	0.1511	0.020*	
C51	0.38367 (19)	0.26988 (18)	0.03145 (17)	0.0149 (5)	
C52	0.4375 (2)	0.34274 (19)	−0.01348 (18)	0.0195 (5)	
H52	0.4234	0.4112	−0.0014	0.023*	
C53	0.5115 (2)	0.3138 (2)	−0.07585 (19)	0.0231 (6)	
H53	0.5484	0.3631	−0.1035	0.028*	
C54	0.5305 (2)	0.2114 (2)	−0.09690 (18)	0.0223 (6)	
H54	0.5781	0.1917	−0.1404	0.027*	
C55	0.4783 (2)	0.13849 (19)	−0.05308 (19)	0.0215 (6)	
H55	0.4915	0.0699	−0.0666	0.026*	
C56	0.4061 (2)	0.16786 (18)	0.01122 (18)	0.0172 (5)	
H56	0.3724	0.1186	0.0412	0.021*	
C61	0.3378 (2)	0.43490 (17)	0.17463 (17)	0.0132 (5)	
C62	0.4539 (2)	0.44975 (18)	0.22122 (17)	0.0155 (5)	
H62	0.5009	0.3988	0.2081	0.019*	
C63	0.4998 (2)	0.53969 (18)	0.28686 (18)	0.0172 (5)	
H63	0.5768	0.5482	0.3185	0.021*	
C64	0.4307 (2)	0.61687 (18)	0.30531 (18)	0.0185 (5)	
H64	0.4615	0.6774	0.3488	0.022*	
C65	0.3157 (2)	0.60363 (17)	0.25872 (18)	0.0171 (5)	
H65	0.2696	0.6557	0.2705	0.021*	
C66	0.2690 (2)	0.51291 (17)	0.19447 (17)	0.0159 (5)	
H66	0.1915	0.5041	0.1645	0.019*	
C71	0.1656 (2)	0.33729 (17)	−0.00414 (17)	0.0144 (5)	
C72	0.1657 (2)	0.43049 (19)	−0.04171 (18)	0.0201 (5)	
H72	0.2205	0.4837	−0.0086	0.024*	
C73	0.0844 (2)	0.4447 (2)	−0.12838 (19)	0.0230 (6)	
H73	0.0841	0.5077	−0.1521	0.028*	
C74	0.0037 (2)	0.3651 (2)	−0.17950 (19)	0.0235 (6)	
H74	−0.0506	0.3745	−0.2374	0.028*	
C75	0.0044 (2)	0.2714 (2)	−0.14401 (18)	0.0214 (6)	
H75	−0.0489	0.2176	−0.1787	0.026*	
C76	0.0843 (2)	0.25779 (18)	−0.05686 (17)	0.0161 (5)	
H76	0.0838	0.1949	−0.0332	0.019*	

### Atomic displacement parameters (Å^2^)

**Table:**

	*U*^11^	*U*^22^	*U*^33^	*U*^12^	*U*^13^	*U*^23^
Ru	0.01137 (10)	0.01228 (9)	0.01083 (9)	0.00130 (7)	0.00208 (7)	0.00070 (7)
P1	0.0119 (3)	0.0124 (3)	0.0115 (3)	0.0009 (2)	0.0017 (2)	0.0017 (2)
P2	0.0116 (3)	0.0131 (3)	0.0105 (3)	0.0014 (2)	0.0019 (2)	0.0014 (2)
O1	0.0226 (10)	0.0171 (9)	0.0299 (10)	0.0043 (7)	0.0076 (8)	0.0006 (8)
O2	0.0134 (9)	0.0197 (9)	0.0259 (9)	0.0021 (7)	0.0073 (7)	−0.0017 (7)
O3	0.0259 (10)	0.0210 (9)	0.0206 (9)	0.0107 (8)	0.0012 (8)	−0.0010 (7)
O4	0.0249 (10)	0.0211 (9)	0.0137 (8)	0.0077 (7)	0.0003 (7)	−0.0053 (7)
O5	0.0104 (9)	0.0228 (9)	0.0218 (9)	0.0017 (7)	−0.0012 (7)	−0.0004 (7)
O6	0.0133 (9)	0.0172 (8)	0.0114 (8)	0.0010 (7)	0.0013 (7)	0.0010 (7)
O7	0.0305 (11)	0.0170 (9)	0.0240 (10)	−0.0009 (8)	0.0078 (8)	−0.0037 (8)
C1	0.0142 (12)	0.0151 (11)	0.0124 (11)	0.0021 (9)	0.0032 (9)	0.0044 (9)
C2	0.0105 (12)	0.0202 (12)	0.0134 (11)	0.0021 (9)	0.0002 (9)	0.0052 (10)
C3	0.0198 (13)	0.0153 (11)	0.0092 (11)	−0.0014 (10)	0.0021 (10)	0.0016 (9)
C4	0.0151 (12)	0.0125 (11)	0.0129 (11)	0.0007 (9)	0.0037 (9)	0.0036 (9)
C5	0.0161 (12)	0.0145 (11)	0.0101 (11)	−0.0003 (9)	0.0030 (9)	0.0049 (9)
C6	0.0151 (13)	0.0154 (11)	0.0146 (12)	0.0015 (9)	0.0046 (10)	0.0009 (9)
C7	0.0124 (12)	0.0209 (12)	0.0141 (11)	0.0012 (10)	0.0032 (10)	0.0080 (10)
C8	0.0132 (12)	0.0208 (13)	0.0131 (12)	0.0021 (10)	−0.0011 (10)	0.0016 (10)
C9	0.0175 (14)	0.0331 (16)	0.0389 (17)	0.0047 (12)	0.0111 (12)	−0.0093 (13)
C10	0.0153 (13)	0.0137 (11)	0.0154 (12)	−0.0027 (9)	0.0038 (10)	0.0004 (9)
C11	0.0376 (17)	0.0237 (14)	0.0183 (13)	0.0090 (12)	0.0056 (12)	−0.0061 (11)
C12	0.0133 (13)	0.0323 (15)	0.0220 (13)	−0.0004 (11)	−0.0031 (10)	−0.0014 (11)
C13	0.0125 (12)	0.0191 (13)	0.0149 (12)	0.0017 (9)	0.0035 (10)	0.0054 (10)
C21	0.0181 (13)	0.0125 (11)	0.0119 (11)	−0.0016 (9)	0.0054 (10)	0.0029 (9)
C22	0.0160 (13)	0.0168 (12)	0.0185 (12)	−0.0005 (10)	0.0055 (10)	0.0022 (10)
C23	0.0206 (14)	0.0226 (13)	0.0142 (12)	−0.0068 (10)	−0.0008 (10)	0.0029 (10)
C24	0.0290 (15)	0.0236 (13)	0.0159 (12)	−0.0053 (11)	0.0080 (11)	−0.0039 (10)
C25	0.0236 (14)	0.0169 (12)	0.0220 (13)	−0.0009 (10)	0.0116 (11)	−0.0006 (10)
C26	0.0185 (13)	0.0148 (11)	0.0154 (12)	−0.0007 (10)	0.0051 (10)	0.0037 (9)
C31	0.0171 (13)	0.0146 (11)	0.0117 (11)	0.0020 (10)	0.0031 (10)	0.0003 (9)
C32	0.0194 (14)	0.0181 (12)	0.0241 (13)	0.0016 (10)	0.0057 (11)	0.0039 (10)
C33	0.0198 (14)	0.0255 (14)	0.0275 (14)	0.0077 (11)	0.0039 (11)	0.0048 (11)
C34	0.0292 (15)	0.0147 (12)	0.0218 (13)	0.0082 (11)	0.0059 (11)	0.0021 (10)
C35	0.0266 (15)	0.0144 (12)	0.0191 (13)	0.0009 (10)	0.0051 (11)	0.0004 (10)
C36	0.0209 (13)	0.0174 (12)	0.0145 (12)	0.0030 (10)	0.0026 (10)	0.0003 (10)
C41	0.0136 (12)	0.0134 (11)	0.0150 (12)	0.0007 (9)	0.0014 (9)	0.0039 (9)
C42	0.0182 (13)	0.0166 (12)	0.0153 (12)	0.0014 (10)	0.0031 (10)	0.0029 (10)
C43	0.0195 (14)	0.0215 (13)	0.0237 (13)	−0.0014 (10)	0.0067 (11)	0.0059 (11)
C44	0.0154 (13)	0.0214 (13)	0.0268 (14)	−0.0044 (10)	0.0007 (11)	0.0044 (11)
C45	0.0191 (13)	0.0181 (12)	0.0176 (12)	−0.0015 (10)	−0.0015 (10)	−0.0001 (10)
C46	0.0180 (13)	0.0153 (12)	0.0173 (12)	0.0008 (10)	0.0051 (10)	0.0047 (10)
C51	0.0105 (12)	0.0217 (12)	0.0110 (11)	0.0019 (10)	0.0003 (9)	0.0007 (10)
C52	0.0216 (14)	0.0207 (13)	0.0164 (12)	0.0030 (10)	0.0043 (10)	0.0027 (10)
C53	0.0222 (14)	0.0321 (15)	0.0175 (13)	0.0020 (11)	0.0077 (11)	0.0076 (11)
C54	0.0147 (13)	0.0370 (15)	0.0148 (12)	0.0045 (11)	0.0039 (10)	0.0004 (11)
C55	0.0192 (14)	0.0225 (13)	0.0202 (13)	0.0048 (11)	0.0022 (11)	−0.0029 (11)
C56	0.0151 (13)	0.0191 (12)	0.0159 (12)	0.0005 (10)	0.0023 (10)	0.0001 (10)
C61	0.0138 (12)	0.0144 (11)	0.0114 (11)	−0.0015 (9)	0.0029 (9)	0.0029 (9)
C62	0.0174 (13)	0.0177 (12)	0.0121 (11)	0.0005 (10)	0.0031 (10)	0.0059 (9)
C63	0.0171 (13)	0.0190 (12)	0.0149 (12)	−0.0034 (10)	0.0027 (10)	0.0036 (10)
C64	0.0232 (14)	0.0154 (12)	0.0162 (12)	−0.0051 (10)	0.0068 (10)	−0.0011 (10)
C65	0.0197 (13)	0.0141 (11)	0.0187 (12)	0.0010 (10)	0.0077 (10)	0.0012 (10)
C66	0.0162 (13)	0.0184 (12)	0.0143 (12)	0.0002 (10)	0.0055 (10)	0.0035 (10)
C71	0.0156 (12)	0.0170 (12)	0.0103 (11)	0.0029 (9)	0.0030 (9)	0.0006 (9)
C72	0.0227 (14)	0.0190 (12)	0.0180 (12)	0.0042 (10)	0.0033 (11)	0.0018 (10)
C73	0.0275 (15)	0.0219 (13)	0.0201 (13)	0.0106 (11)	0.0024 (11)	0.0064 (11)
C74	0.0172 (14)	0.0406 (16)	0.0126 (12)	0.0115 (12)	0.0012 (10)	0.0029 (11)
C75	0.0166 (13)	0.0313 (14)	0.0141 (12)	−0.0006 (11)	0.0032 (10)	−0.0027 (11)
C76	0.0169 (13)	0.0202 (12)	0.0106 (11)	0.0010 (10)	0.0027 (10)	0.0014 (9)

### Geometric parameters (Å, °)

**Table:**

Ru—C13	1.907 (2)	C31—C36	1.400 (3)
Ru—C1	2.038 (2)	C32—C33	1.396 (3)
Ru—C5	2.110 (2)	C32—H32	0.9300
Ru—O6	2.2164 (16)	C33—C34	1.385 (4)
Ru—P1	2.3796 (6)	C33—H33	0.9300
Ru—P2	2.3919 (6)	C34—C35	1.383 (4)
P1—C41	1.833 (2)	C34—H34	0.9300
P1—C21	1.838 (2)	C35—C36	1.394 (3)
P1—C31	1.845 (2)	C35—H35	0.9300
P2—C61	1.832 (2)	C36—H36	0.9300
P2—C71	1.842 (2)	C41—C42	1.397 (3)
P2—C51	1.844 (2)	C41—C46	1.400 (3)
O1—C8	1.216 (3)	C42—C43	1.387 (3)
O2—C8	1.354 (3)	C42—H42	0.9300
O2—C9	1.440 (3)	C43—C44	1.385 (3)
O3—C10	1.214 (3)	C43—H43	0.9300
O4—C10	1.352 (3)	C44—C45	1.385 (4)
O4—C11	1.442 (3)	C44—H44	0.9300
O5—C7	1.341 (3)	C45—C46	1.391 (3)
O5—C12	1.450 (3)	C45—H45	0.9300
O6—C7	1.248 (3)	C46—H46	0.9300
O7—C13	1.154 (3)	C51—C56	1.394 (3)
C1—C2	1.368 (3)	C51—C52	1.404 (3)
C1—C8	1.501 (3)	C52—C53	1.391 (3)
C2—C3	1.435 (3)	C52—H52	0.9300
C2—H2	0.9300	C53—C54	1.389 (4)
C3—C4	1.370 (3)	C53—H53	0.9300
C3—H3	0.9300	C54—C55	1.385 (4)
C4—C5	1.463 (3)	C54—H54	0.9300
C4—C10	1.497 (3)	C55—C56	1.392 (3)
C5—C6	1.363 (3)	C55—H55	0.9300
C6—C7	1.432 (3)	C56—H56	0.9300
C6—H6	0.9300	C61—C66	1.397 (3)
C9—H9A	0.9600	C61—C62	1.399 (3)
C9—H9B	0.9600	C62—C63	1.388 (3)
C9—H9C	0.9600	C62—H62	0.9300
C11—H11A	0.9600	C63—C64	1.387 (3)
C11—H11B	0.9600	C63—H63	0.9300
C11—H11C	0.9600	C64—C65	1.388 (3)
C12—H12A	0.9600	C64—H64	0.9300
C12—H12B	0.9600	C65—C66	1.390 (3)
C12—H12C	0.9600	C65—H65	0.9300
C21—C22	1.394 (3)	C66—H66	0.9300
C21—C26	1.406 (3)	C71—C72	1.393 (3)
C22—C23	1.392 (3)	C71—C76	1.397 (3)
C22—H22	0.9300	C72—C73	1.392 (3)
C23—C24	1.377 (4)	C72—H72	0.9300
C23—H23	0.9300	C73—C74	1.389 (4)
C24—C25	1.390 (4)	C73—H73	0.9300
C24—H24	0.9300	C74—C75	1.386 (4)
C25—C26	1.387 (3)	C74—H74	0.9300
C25—H25	0.9300	C75—C76	1.386 (3)
C26—H26	0.9300	C75—H75	0.9300
C31—C32	1.398 (3)	C76—H76	0.9300
			
C13—Ru—C1	95.87 (9)	C25—C26—H26	119.6
C13—Ru—C5	170.35 (9)	C21—C26—H26	119.6
C1—Ru—C5	91.76 (9)	C32—C31—C36	118.5 (2)
C13—Ru—O6	94.98 (8)	C32—C31—P1	119.50 (18)
C1—Ru—O6	168.84 (7)	C36—C31—P1	121.94 (18)
C5—Ru—O6	77.73 (7)	C33—C32—C31	120.3 (2)
C13—Ru—P1	88.00 (7)	C33—C32—H32	119.9
C1—Ru—P1	93.61 (6)	C31—C32—H32	119.9
C5—Ru—P1	85.63 (6)	C34—C33—C32	120.5 (2)
O6—Ru—P1	89.36 (4)	C34—C33—H33	119.7
C13—Ru—P2	99.24 (7)	C32—C33—H33	119.7
C1—Ru—P2	85.74 (6)	C35—C34—C33	119.7 (2)
C5—Ru—P2	87.18 (6)	C35—C34—H34	120.1
O6—Ru—P2	89.96 (4)	C33—C34—H34	120.1
P1—Ru—P2	172.76 (2)	C34—C35—C36	120.2 (2)
C41—P1—C21	104.31 (11)	C34—C35—H35	119.9
C41—P1—C31	99.28 (10)	C36—C35—H35	119.9
C21—P1—C31	101.54 (10)	C35—C36—C31	120.8 (2)
C41—P1—Ru	114.53 (8)	C35—C36—H36	119.6
C21—P1—Ru	115.29 (7)	C31—C36—H36	119.6
C31—P1—Ru	119.49 (8)	C42—C41—C46	118.5 (2)
C61—P2—C71	104.49 (10)	C42—C41—P1	122.52 (17)
C61—P2—C51	102.69 (11)	C46—C41—P1	118.21 (18)
C71—P2—C51	98.77 (10)	C43—C42—C41	120.9 (2)
C61—P2—Ru	111.47 (7)	C43—C42—H42	119.6
C71—P2—Ru	116.69 (8)	C41—C42—H42	119.6
C51—P2—Ru	120.55 (8)	C44—C43—C42	120.1 (2)
C8—O2—C9	115.36 (19)	C44—C43—H43	120.0
C10—O4—C11	115.26 (19)	C42—C43—H43	120.0
C7—O5—C12	116.16 (18)	C43—C44—C45	119.9 (2)
C7—O6—Ru	110.09 (14)	C43—C44—H44	120.1
C2—C1—C8	114.3 (2)	C45—C44—H44	120.1
C2—C1—Ru	124.54 (17)	C44—C45—C46	120.3 (2)
C8—C1—Ru	120.77 (16)	C44—C45—H45	119.8
C1—C2—C3	127.7 (2)	C46—C45—H45	119.8
C1—C2—H2	116.1	C45—C46—C41	120.3 (2)
C3—C2—H2	116.1	C45—C46—H46	119.8
C4—C3—C2	128.4 (2)	C41—C46—H46	119.8
C4—C3—H3	115.8	C56—C51—C52	118.1 (2)
C2—C3—H3	115.8	C56—C51—P2	122.01 (18)
C3—C4—C5	122.2 (2)	C52—C51—P2	119.59 (18)
C3—C4—C10	117.1 (2)	C53—C52—C51	120.8 (2)
C5—C4—C10	120.7 (2)	C53—C52—H52	119.6
C6—C5—C4	122.0 (2)	C51—C52—H52	119.6
C6—C5—Ru	113.07 (16)	C54—C53—C52	120.0 (2)
C4—C5—Ru	124.71 (16)	C54—C53—H53	120.0
C5—C6—C7	116.0 (2)	C52—C53—H53	120.0
C5—C6—H6	122.0	C55—C54—C53	119.9 (2)
C7—C6—H6	122.0	C55—C54—H54	120.0
O6—C7—O5	120.6 (2)	C53—C54—H54	120.0
O6—C7—C6	123.1 (2)	C54—C55—C56	120.0 (2)
O5—C7—C6	116.3 (2)	C54—C55—H55	120.0
O1—C8—O2	121.8 (2)	C56—C55—H55	120.0
O1—C8—C1	126.9 (2)	C55—C56—C51	121.1 (2)
O2—C8—C1	111.25 (19)	C55—C56—H56	119.4
O2—C9—H9A	109.5	C51—C56—H56	119.4
O2—C9—H9B	109.5	C66—C61—C62	118.7 (2)
H9A—C9—H9B	109.5	C66—C61—P2	121.15 (18)
O2—C9—H9C	109.5	C62—C61—P2	119.62 (18)
H9A—C9—H9C	109.5	C63—C62—C61	120.7 (2)
H9B—C9—H9C	109.5	C63—C62—H62	119.6
O3—C10—O4	121.5 (2)	C61—C62—H62	119.6
O3—C10—C4	126.2 (2)	C64—C63—C62	120.0 (2)
O4—C10—C4	112.3 (2)	C64—C63—H63	120.0
O4—C11—H11A	109.5	C62—C63—H63	120.0
O4—C11—H11B	109.5	C63—C64—C65	119.9 (2)
H11A—C11—H11B	109.5	C63—C64—H64	120.1
O4—C11—H11C	109.5	C65—C64—H64	120.1
H11A—C11—H11C	109.5	C64—C65—C66	120.3 (2)
H11B—C11—H11C	109.5	C64—C65—H65	119.8
O5—C12—H12A	109.5	C66—C65—H65	119.8
O5—C12—H12B	109.5	C65—C66—C61	120.4 (2)
H12A—C12—H12B	109.5	C65—C66—H66	119.8
O5—C12—H12C	109.5	C61—C66—H66	119.8
H12A—C12—H12C	109.5	C72—C71—C76	118.6 (2)
H12B—C12—H12C	109.5	C72—C71—P2	123.32 (18)
O7—C13—Ru	172.5 (2)	C76—C71—P2	117.82 (17)
C22—C21—C26	118.1 (2)	C73—C72—C71	120.6 (2)
C22—C21—P1	120.81 (18)	C73—C72—H72	119.7
C26—C21—P1	120.95 (17)	C71—C72—H72	119.7
C23—C22—C21	120.6 (2)	C74—C73—C72	120.1 (2)
C23—C22—H22	119.7	C74—C73—H73	120.0
C21—C22—H22	119.7	C72—C73—H73	120.0
C24—C23—C22	120.7 (2)	C75—C74—C73	119.8 (2)
C24—C23—H23	119.6	C75—C74—H74	120.1
C22—C23—H23	119.6	C73—C74—H74	120.1
C23—C24—C25	119.6 (2)	C74—C75—C76	120.0 (2)
C23—C24—H24	120.2	C74—C75—H75	120.0
C25—C24—H24	120.2	C76—C75—H75	120.0
C26—C25—C24	120.1 (2)	C75—C76—C71	120.9 (2)
C26—C25—H25	119.9	C75—C76—H76	119.5
C24—C25—H25	119.9	C71—C76—H76	119.5
C25—C26—C21	120.8 (2)		
